# Implementation for Code-Switching from the Perspective of Academic Functions in Teaching English at the MAN 1 Praya

**DOI:** 10.12688/f1000research.177674.1

**Published:** 2026-03-13

**Authors:** Pauzan Pauzan, Edi Sukmojati, Sofyan Hidayat, Jefrianus Alen, Yulius Agustinus Lorenzo Djawa, Wahyuni Lailatul Qomariyah, Nicodemus Dos Reis

**Affiliations:** 1 English Language Education, Universitas Islam Negeri Mataram, Mataram, West Nusa Tenggara, 83116, Indonesia; 2 English Language Education, Universitas Negeri Yogyakarta, Yogyakarta, Special Region of Yogyakarta, 55281, Indonesia; 3 English Language Education, Universitas Negeri Yogyakarta, Yogyakarta, Special Region of Yogyakarta, 55281, Indonesia; 4 English Language Education, Universitas Negeri Yogyakarta, Yogyakarta, Special Region of Yogyakarta, 55281, Indonesia; 5 English Language Education, Universitas Negeri Yogyakarta, Yogyakarta, Special Region of Yogyakarta, 55281, Indonesia; 6 English Language Education, Universitas Negeri Yogyakarta, Yogyakarta, Special Region of Yogyakarta, 55281, Indonesia; 7 Master’s Program in English Language Education, Universitas Pendidikan Ganesha, Singaraja, Bali, 81116, Indonesia

**Keywords:** Implementation, code-switching, academic functions, teaching english

## Abstract

**Background:**

In multilingual educational settings, such as those found in Central Lombok, teachers frequently employ more than one language to facilitate communication. This study explores the phenomenon of code-switching between English and Indonesian during classroom interactions at Madrasah Aliyah Negeri 1 Praya. It examines how this linguistic practice serves specific academic goals and functions within the English language learning environment.

**Methods:**

The researchers utilized a qualitative descriptive approach involving two multilingual English teachers. Data were collected through observation, audio recordings of classroom discussions, and interviews. The study followed three sequential strategic stages: the provision of data, the analysis of data, and the presentation of results. Data were processed using a qualitative descriptive strategy and presented through an informal model involving linguistic explanations of rules.

**Results:**

The findings indicate that code-switching serves two primary academic functions: the delivery of instructional materials and classroom management. Regarding material delivery, teachers used code-switching for twelve purposes, including clarifying information, explaining concepts, checking student comprehension, and providing feedback. For classroom management, the practice fulfilled twelve distinct roles, such as capturing student attention, managing transitions between topics, alleviating tension, and motivating learners.

**Conclusions:**

Code-switching at this institution is an intentional metalinguistic strategy used to bridge communication gaps and achieve academic objectives. The study concludes that the practice is a functional tool in English language instruction that should be utilised if it serves academic goals. However, the results also suggest that the necessity for switching between languages typically diminishes as students’ proficiency in English improves.

## Introduction

Employing two or more languages plays a crucial role in social communication. The practice of using multiple languages during interactions is common. This perspective supports the claim that code-switching frequently occurs among individuals in bilingual and multilingual communities ( Ayu Putu Ari Purniawati et al., 2019). In Lombok, the practice of code-switching occurs when speakers involved in conversation have the ability to use multiple languages. These languages may include regional tongues such as Sasak, Sumbawa, and Bima; the national language, Indonesian; and foreign languages such as English, Arabic, and Japanese. Within different areas of Lombok, people also switch between local dialects, such as Central Lombok and East Lombok, alongside Indonesian and other languages like English. Code-switching refers to the act of shifting from one linguistic variety to another, whether spoken or written, as a way of adjusting communication to fit a specific context ( Novianti & Said, 2021).

Thus, there are several definitions of code-switching: a) code-switching is the event of switching from one language code to another in a speech event ( Manaf, 2021), b) code-switching is the event of switching from one code to another ( Dwinda Khoyriyah et al., 2024), and c) code-switching is the use of another language variation or another language base in a language event as a strategy to adapt to another role or situation or because of the presence of other participants ( Kridalsana, 2008). From these definitions, it can be concluded that code switching is the event of switching from one code to another.

Transfer events can occur in both official and informal settings depending on the circumstances. In various formal settings, such as educational activities at MAN 1 Praya, English teachers routinely move between English and Indonesian to communicate with their students. As a result of the use of many languages by English educators, notably local languages such as Javanese, Indonesian, and English, code-switching occurs. This study aims to examine instances of code-switching within the context of classroom-based English language learning, focusing particularly on the roles these instances serve. The observed code-switching occurred exclusively between English and Indonesian in both directions. Research boundaries were established to enable a more thorough, systematic, and targeted exploration of code-switching phenomena within the instructional setting.

Within bilingualism studies, the concept of 'code-switching' encompasses various synonymous expressions, such as language switching, language alternation, language mixing, and code changing. The expressions code-switching and code-mixing are commonly grouped under the broader term language alternation ( Thohir, 2017). Furthermore, it can be argued that both code-switching and code-mixing are often examined as elements of oral communication, encompassing the shift between various linguistic codes ( Silva, 2018).

The categorization of code mixing can be based on three main criteria: insertion, alternation, and congruent lexicalization. Insertion often appears in societies influenced by colonial histories or migration patterns and involves the integration of lexical items or constituents from one language into the structure of another. Alternation describes the shift from one language to another within a single utterance. This allows the speaker to retain a certain degree of distinction between the languages contained within a clause. The phenomenon known as congruent lexicalization occurs when two languages share a grammatical framework that allows for the substitution of lexical components from either language ( Situmorang et al., 2023). It is possible for the mixing of languages to occur in more than two languages. In addition, the English language may be mixed, which will result in increased uncertainty regarding the meaning of the utterances ( Abidasari & Lestari, 2020). In the context of bilingualism, a phenomenon known as code-switching, language mixing, or borrowing can be observed ( Nurjaleka & Supriatnaningsih, 2021).

In a large number of libraries, the terms “code-switching” and “code-mixing” are frequently used. Regarding other aspects, the majority of linguists favor the use of code-switching and code-mixing over the terminology employed. Code-mixing refers to the amalgamation of two or more languages without altering the subject matter ( Rosmiaty, Ratnawaty, 2020), whereas code-switching involves the alternation between languages within spoken discourse, transitioning from one linguistic code to another during communication; code-mixing, on the other hand, employs multiple codes within a single utterance ( Silva, 2018). Distinguishing between code-switching and code-mixing. Nonetheless, there is a difference between the applications of each method ( Ida Ayu Agung Putri Sistajati & Putu Eka Dambayana Suputra, 2022). Code-switching and mixing are two distinct practices that serve different aims. Code-switching involves switching between sentences in different languages, whereas code-mixing involves incorporating words from a foreign language into the language used locally.

The term “code-switching” was utilized in this study. Code-switching is defined as the transition between English and Indonesian, which is carried out by an English teacher at MAN 1 Praya during interpersonal classroom interactions. Code-switching across languages is a metalinguistic strategy that enables speakers to engage in different language systems to communicate more effectively. Events that involve switching between languages in a school setting are conducted with the goal of doing so. The phenomenon of code-switching fulfills a specific function. Code-switching serves several functions, one of which is communication facilitation. Code mixing denotes an individual's ability to use multiple languages. Individuals employ their proficiency in multiple languages when necessary, or when a specific goal is present ( Yulia et al., 2021). Code-switching can serve as a communicative bargaining tool. Code-switching functions as a mechanism for navigating challenging circumstances in which effective communication is compromised ( Astuti, 2020). Code-switching may serve as a communicative method for a sustainability control act. Code-switching constitutes a linguistic strategy ( Abdelatia et al., 2023). Code-switching serves as a communicative strategy to address language barriers, helping to prevent breakdowns in interaction while also functioning as an indicator of community speech patterns and the identity of particular speakers ( Simatupang & Gunawan, 2023). Code-switching is an interlanguage communication strategy ( Fauziati Penerbit, Arie Frinola Minovia, Resti Yulistia Muslim, 2021).

Eleven primary functions of code-switching are employed by Spanish learners of English as a second language. These are (1) representing speech, (2) quoting or imitating, (3) accommodating conversational turns, (4) changing the topic, (5) switching language due to situational factors, (6) expressing insistence, and (7) emphasizing particular ideas. Moreover, code-switching can also serve to (8) clarify or persuade, (9) specify meaning, (10) shift the form of a question, and (11) mark discourse boundaries. In addition, an alternative perspective highlights 11 pedagogical applications of code-switching in multilingual educational contexts: (1) clarifying instructional objectives, (2) delivering directions effectively, (3) facilitating linguistic communication, (4) promoting social interaction, (5) demonstrating language competence, (6) signaling changes in subject matter, (7) showing fluency in communication, (8) emphasizing the significance of the topic, (9) assessing comprehension levels, (10) repeating key information, and (11) fostering a sense of unity among learners ( Fachriyah, 2011).

There are three distinct categories of function. As can be seen in the following evidence: a) To facilitate access to the curriculum and development of academic knowledge, it is necessary to assist students in comprehending the material. b) In the context of classroom administration, this means fostering discipline and suggesting changes in the educational environment. c) Humanizing classroom settings and making it easier for students to negotiate their identities are two aspects of interpersonal connections that are considered ( Hafid & Margana, 2022). There are four domains of educational material: introducing concepts, reviewing prior lessons, engaging learners' attention, and providing praise ( Ali, 2021). While the term “code-switching” refers to the process of moving from one language to another inside a sentence by utilizing the grammatical units of phrases, clauses, or words, that is not the only sort of code-switching ( Leo & Sudarmawan, 2022).

In the context of English language learning in the classroom, code-switching serves a number of functions, one of which is the academic goal. Two categories can be used to classify academic functions: (a) the provision of materials that are relevant to the subject matter, and (b) classroom management of the classroom ( Simatupang & Gunawan, 2023). At MAN 1 Praya, these two functionalities are utilized to analyze code-switching occurrences that occur during classroom communication between teachers and pupils.

## Methods

In this study, the research approach utilized was descriptive and qualitative. Qualitative research is characterized by its descriptive content and often uses analytical approaches. This qualitative descriptive study used three different phases of strategy in sequential order ( Sugiyono, 2018). There are three stages: (1) provision of data, (2) analysis of the data, and (3) presentation of the study’s results. To gather information, this study employed methodological procedures that involve observation, in addition to recording and interviewing techniques. The utilization of all languages in English instruction, as well as the adoption of code-switching by the English teachers at MAN 1 Praya in classroom conversation, were both evaluated during the period of observation. To make these observations more accessible, recording techniques were utilized and the recordings were transcribed. In the course of this investigation, listening strategies were investigated in conjunction with note-taking procedures. The primary focus of this investigation is on recorded forms of speech, which include communication in the classroom, as well as instances of language transfer between English and Indonesian. A qualitative descriptive strategy was utilized during the research project that involved the processing of data. In addition, the presentation of data makes use of an informal model, namely a presentation model that is articulated through language explanations of rules. Each analysis provides a comprehensive and precise description of the informal presentation paradigm.

This study examined two multilingual English educators teaching at MAN 1 Praya in Central Lombok. It is presumed that these instructors engaged in translations between English and Indonesian during their English lessons. The dataset in this research consists of spoken discourse that illustrates instances of code-switching between the two languages. The investigation was conducted by English teachers at MAN 1 Praya, using contextual data drawn from classroom interactions led by these instructors. The speech samples, containing both English and Indonesian segments, were documented along with relevant contextual details, and categorized according to the teacher’s initials, gender, and grade level (junior, middle, and senior high school). However, regarding ethical procedures, informant consent was provided in writing by the participants. A sample consent form has been included as Extended Data to document this process. This written consent ensured that participants were fully aware of the research purpose, the voluntary nature of their participation, and their right to withdraw at any time without consequences. The primary aim of this research was to identify the possible challenges inherent in classroom discourse. Data sources included a combination of observational field notes and conversation transcripts obtained from the recorded classroom sessions. The analysis was limited to identifying and interpreting code-switching events performed by English teachers at MAN 1 Praya during classroom discussion.

## Results and discussion

The code-switching function present in interactional communication serves as an academic goal that aims to facilitate learning. The material learning function and the class administration function are the two sorts of functions that fall under this category. The findings are described as follows.

### Function of submitting english learning materials

This material fulfills several purposes, such as: (1) clarifying information; (2) reiterating key points; (3) explaining concepts; (4) exploring ideas; (5) elaborating on details; (6) checking comprehension; (7) highlighting specific linguistic elements; (8) drawing inferences; (9) developing vocabulary; (10) addressing students’ assignments or work; (11) delivering feedback; and (12) engaging in reflection. A description of each purpose is provided below:

1. Clarification is a part of this function. On the other hand, clarification refers to the process of making a statement or idea more understandable ( Kääntä & Kasper, 2018). English teachers intend to use code-switching between English and Indonesian during lessons to clarify or validate the questions, responses, or information that students supply. The following illustration illustrates the data for the clarification function, which are highlighted in bold.

S: Peserta didik menyimak penjelasan guru (The students attend the teacher’s explanation).

T: Jika teks naratif dan deskriptif ini kita kaitkan dengan tenses, tense apa yang sesuai untuk masing-masingnya? (When these narrative and descriptive texts are associated with grammatical tenses, which tense is appropriate for each of them?).

S: (Peserta didik belum memahami maksud pertanyaan guru) (The students have not yet comprehended the teacher’s questions).

T: Who knows? If we connect narrative and descriptive texts with tenses, is tense appropriate for narrative and descriptive texts?

S: (Peserta didik tampak tetap belum memahami pertanyaan guru) (The students still appeared to have not comprehended the teacher’s question).

T: Do you understand my question?

S: We do not understand, sir.

T: **One of the reasons I am asking this question is to determine which tense is suitable for narrative and descriptive writing. What is the difference between descriptive text for the present tense and narrative writing for the past tense *? Jadi begitu maksud pak guru*** (So that is what you mean, teacher).

(P/M/XII)

2. A function that involves repetition. The act of repeating words or phrases in the following statement is known as repetition ( Bobihoe et al., 2025). Educators sometimes participate in code-switching between English and Indonesian when teaching English as a Second Language. This is done to restate or repeat word forms, phrases, or clauses while speaking. The purpose of repetition is to either accentuate particular linguistic elements or to unify the meanings of two different forms of language, which ultimately helps pupils have a better understanding of the material. Data that serve as an illustration of the reiteration function are provided.
  T: **OK, now let's discuss conditional sentence. Conditional sentence *adalah kalimat pengandaian yang digunakan untuk mengungkapkan sebab akibat atau kemungkinan terjadinya suatu hal*** *.* (A conditional sentence is a hypothetical construction used to express causality or the possibility of an event occurring.)
  S: (Siswa nampak serius mendengarkan penjelasan gurunya) (The students appeared to listen attentively to the teacher’s explanation).
  T: Itulah Pengertian ***conditional sentence*** anak-anak. Berikutnya saya akan berikan beberapa contoh. Tolong diperhatikan ya. (That is, in the definition of a conditional sentence, students. Next, I provide several examples. Please pay close attention to) issue.

S: Ya pak guru (Yes, Sir)

(F/M/XI)

3. This is the purpose of the explanation. Specifically, it refers to the specifics or explanations that someone provides to make something understandable or obvious ( Páez, 2019). Elucidating the notions or meanings of English forms or clarifying English grammatical norms are the two purposes for the phenomenon of code-switching from English to Indonesian. Students can deepen their understanding of the material by moving between different codes, which enables them to apply what they have learned in a variety of English contexts. Some examples of the data for this function are presented here.

T: Just now we discussed descriptive text.

Selanjutnya, kita akan membahas mengenai simple present tense, yang memiliki keterkaitan dengan teks deskriptif. (Next, we discuss the simple present tense, which is closely related to descriptive text).

S: Siswa serius mendengarkan penjelasan gurunya. (The students listened attentively to the teacher’s explanation.)

T: Simple present tense adalah bentuk keterangan waktu yang menyatakan suatu kejadian yang berlangsung di masa sekarang. The Dapat didefinisikan juga bahwa **simple present tense is used to refer to events, actions, and conditions that are happening all the time, or exist now.** Siapa kira-kira yang bisa bikin contohnya? (The Simple present tense is a temporal form used to express events that occur in the present. It can also be defined as follows: simple present tense is used to refer to events, actions, and conditions that are happening all the time or exist now. Who would like to provide an example?).

S: Saya pak guru (I will, Sir).

T: Silakan (Please go ahead).

S: He read a book.

T: It is not correct. **You should add “s”. So the correct word is “reads *”. Jadi seharusnya kamu menambahkan “s” di kata* “read” *sehingga menjadi* “reads”, because the subject is the third person singular**. Pada kata kerjanya ditambahin “s” itu disebabkan karena subjeknya orang ketiga tunggal. (The addition of “-s” to the verb occurs because the subject is in third-person singular form).

(P/M/XII)

4. This is a function of exploration. Learning acquired through concerted variation, purposeful experimentation, and play is what we mean when we talk about exploration ( Koryak et al., 2018). The English teacher's code-switching from English to Indonesian during instruction elicits prior information pertinent to the English subject matter being covered. The exploratory role of code-switching is illustrated in the data.
  T: ***Baiklah, anak-anak *, (All right, students) just now we discussed tense in the form of the simple present tense. Now we continue discussing the next tense, namely the simple past tense, so that we know more about tenses. *Tolong diperhatikan ya anak-anak mengenai* tense *berikutnya supaya kalian tahu lebih mendalam mengenai tenses tsb.* (Please pay attention, students, to the next tense so that you may gain a deeper understanding of it).**

S: Ya pak guru. (Yes, Sir)

(F/M/XI)

5. This is the role of elaboration. Having the ability to develop ideas in addition to specifics is what is meant by the term “elaboration” ( Handayani et al., 2021). Code-switching from English to Indonesian in the classroom serves to enhance pupils’ comprehension of the existing English course material. The following is a sample of the data for the elaboration function:
  T: Pada kesempatan ini, kita akan bahas tentang conditional sentences (on this occasion, we will discuss the conditional sentence). As expected, this conditional sentence is used to state something that may or may not happen.
  Sebelum kita membahas lebih jauh mengenai Conditional sentence ini, seperti contoh, dan lain sebagainya. ***Siapa yang tahu contohnya?. Silakan angkat tangan,*** (Before we go further into conditional sentences, such as examples and other aspects, **who knows an example? Please raise your hand) who knows to make a sentence?.**

S: Saya pak guru, mau membuat contohnya. (I would like to provide an example, Sir).

T: Sialakan. ***Siapa lagi mau membuat contohnya. Ayo,* (Please go ahead. Who else would like to give an example? Come on) who else wants to make an example?**

(F/M/XI)

6. The purpose of this function was to revise previously taught material. Teachers have a responsibility to be aware of the capabilities of the students, as well as the extent to which the pupils comprehend the content that has been presented to them ( Sujariati et al., 2016). Educators can pose a question and then acquire an answer from the subject, which has been explained in more detail. The transition from English to Indonesian that the English teacher makes during English education serves the purpose of evaluating students' ability to remember the information being presented. Note the punctuation highlighted in the accompanying data section.
  T: Baiklah, anak-anak (all right students) **at this meeting, we are still talking about the subject that we discussed yesterday. What were some of the subjects covered at the meeting that took place yesterday? *Bagaiman, apa ada yang tahu, topik apa yang dibahas pada pertemuan kemarin?*** (So does anyone know what topic was discussed in the previous meeting?).

S: Conditional sentence

T: Ya betul (That is right), does anyone know how to create an example of a conditional sentence?

(P/M/XII)

7. In the context of language, the role is to highlight particular linguistic characteristics or elements of language ( Li et al., 2022). In this setting, students studying English in the classroom come across specialized or technical vocabulary in English, the meanings of which they have not yet become familiar with. Using code-switching between English and Indonesian to emphasize specific vocabulary is a strategy adopted by English teachers to prevent pupils from experiencing difficulties in comprehending certain forms. These functions are highlighted in bold font.
  T: Untuk diketahui bahwa teks naratif berkaitan tense yakni. (It should be noted that narrative texts are related to the tense, namely) **simple past tense**. **Past tense** is a tense shows actions that occurred in the past and were completed at a certain time in the past. Kalau dalam bahasa Indonesianya p **ast tense** adalah **tenses** untuk menunjukkan aksi yang terjadi di masa lampau dan telah selesai pada waktu tertentu di masa lampau. Beberapa contoh **past tense**. (In Indonesian, the past tense refers to a tense used to indicate actions that occurred in the past and were completed at a specific time in the past. Here are several examples of the past tense).
  S: (Semua siswa memperhatikan penjelasan gurunya. (All students paid attention to the teacher’s explanation).

(P/M/XII)

8. From this function, inferences were drawn. Inference-making skills are essential components of student learning and should not be overlooked ( Teo Tang Wee, Goh Jonathan Wee Pin, Aye, 2018). The English teacher employed code-switching from English to Indonesian in the classroom to effectively conclude explanations, ensuring that pupils comprehend the imparted information. These functions are shown in bold inside this data.
  T: Pada pertemuan kali ini, kita akan membahas materi tentang **past tense**. **Simple past tense** merupakan bentuk tense yang digunakan untuk menyatakan suatu perbuatan atau kejadian yang terjadi di masa lalu dan telah selesai pada waktu tertentu di masa tersebut. Dalam bahasa Indonesia, **simple past tense** berarti bentuk tense yang digunakan untuk menggambarkan aksi yang sudah terjadi dan berakhir pada periode waktu tertentu di masa lampau. Polanya adalah **S + V2 + Object**. Contoh Kalimatnya: **I studied English.** Perlu diperhatikan bahwa kata kerja yang digunakan pada kalimat positif adalah **Verb 2**. Namun, apabila membuat kalimat tanya atau kalimat negatif, kita perlu menambahkan kata bantu **“did”**.
  Maksud saya, pada kalimat positif bentuk lampau, kata kerja yang digunakan adalah **verb 2**. Namun, ketika membuat kalimat tanya atau kalimat negatif, kita perlu menambahkan kata bantu **“did”**, misalnya pada kalimat **“Did you study English”** atau **“I didn’t study English”**. Dalam pembuatan kalimat tanya maupun negatif, bentuk kata kerja yang digunakan adalah **verb 1**. (In this *meeting,* we will discuss the material of **past tense**. *Simple past tense* is a form of tense **used to express an action or event that happened in the past** and **was completed at a certain time in the past**. In Indonesian, *simple past tense* means a tense is **used to describe an action that has already happened and ended in a specific period of time in the past**. The pattern was **S + V2 + Object**. Example sentence: *I studied English.* It should be noted that the **verb** used in the positive sentence was **Verb 2**. However, when making an interrogative or negative sentence, we need to add the auxiliary **“did.”** What I mean is that in positive past tense sentences, the **verb** used is **verb 2**. However, when making interrogative or negative sentences, we need to add the auxiliary **“did,”** for example in the sentence *“Did you study English”* or *“I didn’t study English.”* In forming interrogative and negative sentences, the form of the **verb** used was **verb 1).**
  Regarding the past tense, we must have a solid understanding of the definition, the formula, and the examples. Additionally, we must be familiar with the auxiliary verbs that are used in negative statements and interrogative sentences. *Maksud saya, intinya adalah past tense ini perlu kita pahami definisi, rumus, contoh-contonya, dan kita perlu tahu juga kata kerja bantu untuk klimat tanya dan kalimat negatif.* (What I mean is that we need to understand the definition, formula, and examples of the past tense, and we also need to know the auxiliary verbs used for interrogative and negative sentences).
  S: Siswa dengan serius memperhatikan penjelasan gurunya. (The students listened attentively to the teacher’s explanation.)

(P/M/XII)

9. The growth of vocabulary was effective. Instructors teach synonyms to help students increase their vocabulary skills ( Baltayeva, 2024). In English language instruction, certain educators employ code-switching between English and Indonesian to enhance their vocabulary acquisition. The functions are shown in bold.
  T: Selain membahas definisi, rumus, serta contoh penggunaan **simple present tense**, kita juga akan mempelajari sinonim dan antonim dari kata kerja yang digunakan dalam tense tersebut. Sinonim adalah kata yang memiliki makna sama atau hampir sama dengan kata lain, sedangkan antonim adalah kata yang memiliki arti berlawanan dengan kata lainnya. Misalnya, kata kerja **help** memiliki sinonim **assist** dan antonim **hinder**. (In addition to discussing the definition, formula, and examples of the use of the simple present tense, we also study the synonyms and antonyms of the verbs used in this tense. A synonym is a word that has the same or nearly the same meaning as another word, whereas an antonym is a word that has the opposite meaning. For example, verb help has synonym assist and antonym hinder). **What is the synonym of “try”?.**

S: attempt

T: Okay, your answer is correct. **What is the antonym of “try”?.**

S: I do not know, sir.

T: refrain

(P/M/XII)

10. The purpose of deliberating on pupils' coursework or tasks. As part of the English curriculum, teachers typically talk about customary exercises or assignments that students have completed. The training approach is widely utilized by educators to effectively develop the abilities of students, enabling them to competently participate in learning activities associated with the subject matter being studied ( Syarifah & Emiliasari, 2019). The English teacher employed code-switching between English and Indonesian to facilitate student comprehension of the activities being addressed. These functions are shown in bold inside this data.
  T: Kita sudah bahas tentang simple past tense. **Now there is an exercise or assignment to do *. Anak-anak, s*** ***ekarang ada latihan atau tugas yang kalian harus kerjakan**.* (We have already discussed simple past tense. **Now there is an exercise or assignment to do. Students, here is the practice task that you need to complete)**

S: Ya pak guru. (Yes, Sir).

T: Fill in blanks with correct verb forms.

S: Siswa mengerjakan latihan sesuai perintah guru. (The students completed the exercise according to the teacher’s instructions).

T: Have you finished doing it?

S: Ya, sudah pak guru. (Yes, we have finished; Sir).

T: **Bages, answer number 1.**

S: Was

T: **Yes, it is correct.**

(P/M/XII)

11. The function of delivering feedback. Feedback is a reiteration that depends on the outcomes of the student's performance. Feedback constitutes information pertaining to the reciprocal relationship between educators and learners ( Nurachman & Irawan, 2020). Students need to be given feedback on their English learning activities so that they can comprehend both the tasks they have completed successfully and the mistakes they have made in completing the assignments given to them by their instructor. To guarantee that all of the students are able to comprehend the feedback provided, the English teacher will move between English and Indonesian. A sample of data relevant to this function will be found.
  T: Tadi kita sudah mempelajari tentang teks naratif. (Earlier, we learned about narrative text). **Do you get the idea of narrative text? Apakah kalian sudah memahami teks naratif itu? (Have you understood the narrative text?) So, you need to know the definition of narrative text. Jadi, memahami definisi adalah hal yang sangat mendasar, anak-anak (So, understanding the definition is very basic). When you know the definition, you will also know how to create examples. Sekarang saya mau bertanya (Now, I would like to ask a question), what is a narrative text and can you give an example?**

S: Saya pak mau jawab (Sir, I would like to answer).

T: Ya kamu silakan dijawab. (Yes, please go ahead.)

(P/M/XII)

12. This function was used for the reflection. Reflects by directly inquiring about recently acquired information and the means by which it can be obtained ( Hartmann et al., 2023). Instructors are encouraged to confront their own teaching practices, question their own beliefs about teaching and learning, and investigate not only what works in the classroom but also why. Reflection in the classroom helps instructors do all of these things ( Bawaneh et al., 2020). Educators and students contemplate activities related to English language acquisition in English language classes. The English teacher employed code-switching from English to Indonesian to facilitate students' expression of their reflections and comprehension of those shared by the teacher and peers. The function of reflection is emphasized in bold in the data.
  T: Tadi kita telah membicarakan tentang **recount text**, mulai dari pengertian hingga contoh-contohnya. Selanjutnya, saya memiliki beberapa pertanyaan untuk mengetahui apakah kalian memahami penjelasan saya sebelumnya atau tidak. **Berkaitan dengan materi yang baru kita bahas, adakah di antara kalian yang tahu? (**Earlier, we discussed recount text, starting from its definition to several examples. Next, I asked a few questions to determine whether you understood my previous explanation. **In relation to the material we have just covered, is there anyone who knows?). Do any of you know what a recount text means?**

S: Saya pak (Me, Sir).

T: Silakan. (Please go ahead)

S: Recount text is **recount text *adalah jenis teks bahasa Inggris yang menceritakan kembali kejadian atau peristiwa di masa lampau.* (is a type of English text that recounts events or experiences from the past).**

(P/M/XII)

### Class management function

The class management function pertains to the code-switching mechanism designed to regulate the class. This function comprises 12 distinct components: (1) capturing students' attention, (2) assigning tasks, (3) signaling a topic transition, (4) soliciting student assistance, (5) alleviating tension, (6) enforcing student discipline, (7) fostering student motivation, (8) acknowledging student efforts, (9) issuing student warnings, (10) facilitating student participation, (11) reprimanding students, and (12) nurturing interpersonal relationships. Many instances of code-switching functions for classroom management are presented below.

1. To capture students' attention, code-switching between English and Indonesian was employed to capture students' attention, ensuring their focus on the instructional content being delivered. The following exemplifies the data for this function, as indicated in bold font in this dataset.

T: Attention please!

S: Ya pak guru. (Yes, Sir).

T: Sebelum kita mulai belajar (Before we begin the lesson). I want to call your names alphabetically to determine who is present or not.

S: (Siswa memperhatikan instruksi guru dengan serius). (The students paid close attention to the teacher’s instructions.)

T: **Please do not make any noise, pay attention and listen!. *Yang lain tunggu giliran namanya dipanggil ya? (The rest of you, please wait until your names are called, all right?).***

S: (Beberapa siswa menjawab, “Ya pak guru.”) (Several students responded, ‘Yes, Sir.’).

(P/M/XII)

2. Code-switching between English and Indonesian is used to communicate instructions, activities, or assignments to students. This allowed the assignment of tasks. Code-switching is implemented to ensure that students comprehend the actions or tasks that their classmates are carrying out during the process of teaching and learning. The following is an example of a function's data.
  T: **Next, I will give you an assignment in the form of an exercise, please do it**. ***Tolong kerjakan latihan ini ya?.*** Silakan kalian menyusun beberapa kalimat sesuai ketentuan. Kerjakan mengikuti rumus yang telah diberikan. Setelah selesai, saya akan meminta kalian maju untuk menuliskan hasilnya di papan tulis. Do you get what I mean? (Please compose several sentences in accordance with the guidelines. Perform the task by following the formula provided. After finishing, I ask you to come forward and write your results on the board. Do you get what I mean?).

S: Ya pak guru (Yes, Sir).

T: Tolong jika ada yang belum jelas cara membuat kalimat tersebut, tanyakan kepada saya. (If there is anything unclear about how to construct these sentences, please ask me.)

S: (Siswa menjawab, “Baik pak guru.”) (The students responded, “All right, Sir.”)

(P/M/XII)

3. This indicates a shift in subject matter. The English teacher employed code-switching between English and Indonesian to signify a shift in the topic. By altering the codes, students were made aware of the subject under examination. The role of code-switching in indicating a change in topic is shown in bold font in the data.
  T: Previously, we had discussed the first topic. **We continue to discuss the next topic *. Anak-anak mari kita fokus untuk membahas topik kedua ini. (Children, let us focus on discussing this second topic).***

S: (Semua anak menjawab, “Ya pak guru.”) (All the students responded, “Yes, Sir.”).

T: Baiklah anak–anak (All rights, children, and pay attention).

(P/M/XII)

4. The purpose of soliciting assistance from students. English professors engage in code-switching between English and Indonesian to solicit assistance from pupils, such as retrieving textbooks, erasing whiteboards, or opening and closing doors. The bold wording in this data illustrates the role of code-switching in soliciting assistance from the pupils.
  T: ***Wah,, hawa di ruang kelas ini cukup panas (Wah., the air in this classroom is quite hot).* Please, open the window or door *.***
  S: (Siswa menunggu perintah dari gurunya untuk membuka pintu atau jendela) (The students waited for the teacher’s instruction to open the door or window).

T: ***Indra tolong ya dibuka pintunya***. **(Indra, please open the door).**

S: Baik pak guru. (Yes, Sir)

T: ***Tolong juga dihapus papan tulisnya,* before we study** (Please also erase the whiteboard before we study).

S: (Siswa menunggu perintah guru untuk menghapus papan tulis) (The students waited for the teacher’s instruction to erase the whiteboard).

T: Zaki

S: Ya. (Yes).

(P/M/XII)

5. The function of alleviating tension. The persistent use of English by educators in classroom interactions induces stress in certain students with insufficient English proficiency. To alleviate this tension, English teachers transitioned from English to Indonesian. The highlighted text in this data illustrates the role of code-switching in alleviating tension.
  T: I hope that you understand what I say and what I have said. Thus, this learning is effective–that is, my hope. So, do you agree? Why are you silent? Please answer, so this is clear. Why are you silent? Do you understand the statement I have made?
  S: (Siswa kelihatannya tidak paham atas apa yang disampaikan atau dibicarakan gurunya. Sehingga, siswa menjadi tegang). (The students seemed not to understand what the teacher had said, which made them feel tense.)

T: Paham nggak tadi apa yang saya bicarakan (Did you understand what I just explained?).

S: Tidak paham pak guru (No, Sir).

T: ***"Maksud saya tadi adalah saya berharap kalian memahami apa yang telah maupun akan saya sampaikan, sehingga pembelajaran ini dapat berlangsung efektif. Itulah harapan saya." (What I meant earlier was that I hope you understand what I have presented and will present, so that this lesson can proceed effectively. That is my expectation).***

S: Itu maksudnya pak guru (So, that is what you mean, Sir).

T: Ya (Yes).

S: (Siswa kelihatan tidak tegang lagi) (The students no longer appeared tense.)

(P/M/XII)

6. Inspired by students, code-switching between English and Indonesian was employed to motivate pupils to meaningfully engage with the English curriculum. Observe the text emphasized in the data.
  T: I inform you that today I will share the test results. Cuman sayang ada beberapa siswa yang nilainya jelek (Cuman sayang ada beberapa siswa yang nilainya jelek).

S: Wow.

T: Yang nilainya jelek, jangan berkecil hati (for those who received low scores) was not discouraged. The most important thing is that) **you study more seriously and more diligently**. Sekali lagi (one more): **You study more seriously and diligently, okay?**. Tidak ada yang sulit jika kitasungguh-sungguh dan rajin (Nothing is difficult if we are earnest and diligent).

S: (Kelihatannya, siswa mendengarkan motivasi dari gurunya dengan dengan sungguh- sungguh). (The students appeared to listen attentively to their teachers’ motivation.)

(P/M/XII)

7. The responsibility of allowing children to express thanks. Code-switching from English to Indonesian is common practice among English teachers. This is done to recognize the achievements of the pupils in accomplishing the tasks that have been set for them. As a result of the fact that the message conveyed by the English instructor is understandable to all the students in the class, students feel a greater sense of pride in their achievements when they are able to switch from English to Indonesian.
  T. I asked you to answer my questions. Who knows the definition of the simple present tense?

S. Saya tahu definisinya pak guru (I know the definition, Sir).

T. Silakan dijawab Arman (Please answer, Arman).

S. **Simple present tense** adalah bentuk *tenses* yang digunakan untuk **menyatakan kejadian yang terjadi secara teratur, rutin, atau biasa dilakukan pada masa kini** (The simple present tense is a form of tense used to express actions that occur regularly, habitually, or customarily at the present time).

T. **Your answer is very good, Arman, *jawaban yang bagus.* (That is an excellent response).**

(P/M/XII)

8. The Act of Providing Children with Warnings or Information. During the process of learning English, it is noted that certain students arrive late, cause disruptions, demonstrate disruptive conduct to attract attention, delay completing tasks that have been allocated to them, and commit errors, among other problems. To solve this problem, the English instructor was able to transition between English and Indonesian. The following bold language explains the function that code-switching plays in the process of providing warnings to students.
  T: Yesterday, there was the homework that I gave you.

Apa kalian semua sudah mengerjakannya? (Have you finished working on it?).

S: Sudah pak. (Yes, Sir)

T: Kalau begitu (In that case), I want to check the homework to find out whether you have all done it or not.

S: (Siswa kelihatan ketakutan) (The students appeared frightened).

T: Wah, setelah saya cek (Well, after I checked). It turns out that some of you do not do their homework.

***Jangan malas ya, mengerjakan tugas.*** Sekali lagi saya sampaikan, jika diberikan tugas oleh pak guru, (Do not be lazy in doing your assignments. Once again, I remind you if the teacher gives you an assignment). **Don't be lazy, do it and do it.**

S: (Kelihatannya ada siswa yang ketiduran di dalam kelas) (it seems that there are students who fell asleep in class.)

T: Bages, Bages bangun-bangun. Bages: Why did you fall asleep in the classroom early in the morning?

Cuci muka sana. ***Jangan banyak begadang makanya, biar tidak ngantuk di dalam kelas.*** Tolong dengar saya (Bages, Bages, wake up. Bages: Why do you fall asleep in the classroom so early in the morning? Go wash your face. That is why you should not stay up too late, so you will not feel sleepy in class. Please listen to me). **Don't stay up too late, so you can follow your studies well *.***

S: Ya pak. (Yes, Sir).

(P/M/XII)

9. By allowing pupils to take turns. Code-switching occurs when English teachers switch from English to Indonesian and vice versa when they are giving turns to answer questions or conduct other responsibilities. In the data displayed here, the function of code-switching, which occurs when pupils are given turns, is highlighted in bold.
  T: Now I ask you to answer my question regarding what I have explained earlier. Bagaimana, apa kalian siap? (Well, are you ready?).

S: Ya pak guru (Yes, Sir).

T: Siapa yang tahu? (Who knows?). What is a narrative text?

S: Saya pak (Me, Sir)

T: Ya, Majid coba dijawab (Yes, Majid, please answer).

S: Narrative text adalah sebuah teks yang menceritakan rangkaian suatu peristiwa secara berurutan dan saling terhubung satu sama lain. (A narrative text is a type of text that tells a sequence of events in order, with each event connected to others).

T: **Wow, amazing, your answer is very good.**

S: Thank you, Pak Guru (Sir).

(P/M/XII)

10. Scolding students. When learning English, students often become angry or irritated with English teachers. Thus, the message of anger or annoyance is conveyed to students, and English teachers tend to switch codes from English to Indonesian. See the following example.
  S: (Ada seorang siswa bernama Ali. Dia selalu berisik pada saat berlangsungnya kegiatan belajar mengajar di kelas) (There was a student named Ali. He was always noisy during the teaching and learning process in class).

T: (Guru marahin Ali) (The teacher scolded Ali).

S: (Pada saat Ali mendengar gurunya marah, pandangannya menuju gurunya) (When Ali heard his teacher scolding him, he turned his gaze toward the teacher).

T: **Shut up Ali**; we are learning. Kita lagi belajar ini. Diam ya, jangan berisik (We are now in the middle of the class. Be quiet, do not make noise).

S: Maaf pak (Sorry, Sir).

(P/M/XII)

11. The function of teasing students. The incidence of code-switching from English to Indonesian or vice versa in classroom communication is intended to insinuate students who do not pay attention to the explanations given. The following is an example of data related to this function:
  T: Okay, on this occasion, I will explain descriptive text.
  Mohon diperhatikan, anak-anak. Hal yang akan saya jelaskan terutama mencakup pengertian, karakteristik, dan contoh-contohnya (Please pay close attention, children. What I am going to explain mainly concerns definitions, characteristics, and some examples).
  S: (Ada beberapa siswa yang tidak serius mendengarkan penjelasan gurunya) (Several students appear not to be listening attentively to the teacher’s explanation).
  T: He, he, those are some people at the back; they do not seem to be seriously listening to my explanation. ***Dasar IQ jongkok, nilai merah di rapornya selalu di atas tiga (it seems your IQ is quite low; your report card shows you consistently score above three in failing grades).***

S: (Siswa yang disindir diam seribu bahasa) (The student remains silent, saying nothing in response.)

(P/M/XII)

12. Maintaining personal relationships in a good standing. The maintenance of interpersonal relationships between teachers and students is accomplished through the use of code-switching events in English language learning in the classroom. These events switched from English to Indonesian. The following is an example of data that can be used with this function.
  T: *What about it? Is it clear to you to understand?*

S *: Yes*

T: **Okey, Ardi**. *Let's go on for the next material.*

T: Discussing crime is very interesting this morning. Is that right? **Ardi**?

S: *Yes.*

(P/M/XII)

## Conclusion

Two academic functions are served by the code-switching phenomenon between English and Indonesian that English teachers utilize during teaching. These functions are the transmission of content and facilitation of classroom management. Twelve distinct functions were associated with the distribution of the materials. The following are the functions that are included: (1) clarification, (2) reiteration, (3) explanation, (4) exploration, (5) elaboration, (6) verification of understanding, (7) emphasis on particular linguistic elements, (8) generation of inferences, (9) enhancement of vocabulary, (10) discussion of participants' assignments or educational work, (11) provision of feedback, and (12) reflection. The function of class management is comprised of twelve distinct components, which are as follows: (1) attracting the attention of students; (2) providing direction; (3) indicating a transition from one topic to another; (4) requesting assistance from students; (5) reducing tension; (6) enforcing student discipline; (7) motivating students to work hard; (8) recognizing student efforts; (9) issuing warnings to students; (10) facilitating student participation; (11) reprimanding students; and (12) cultivating interpersonal relationships. In the context of English language learning in the classroom, the findings suggest that the phenomenon of code-switching between English and Indonesian is not something that should be contested. This is because the English teachers at MAN 1 Praya intentionally switched between several languages to communicate with their students. The practice of switching from English to Indonesian in the context of English language instruction ought to be conveyed to English teachers, provided that switching is carried out in a manner consistent with the functions of the language.

## Ethical approval statement

This research was reviewed and approved by the Research Ethics Committee of Universitas Islam Negeri Mataram, Indonesia. The ethical clearance was granted under Ethics Approval Number: UIN.02/KEP/2023/001 (Approval Letter Number: UIN.02/KEP/2023), issued on February 10, 2023. The approval permitted the researcher to conduct the study using a survey method. All research procedures were conducted in accordance with the ethical standards of the institutional research ethics committee.

## Data Availability

The datasets generated and/or analyzed during this research are not publicly available due to ethical constraints and the need to protect participant confidentiality. The Ethics Review Board (IRB) of State Islamic University of Mataram has reviewed and approved the research protocol, including restrictions on open data sharing. Access to the data may be granted upon reasonable request to pauzanharis@uinmataram.ac.id, subject to IRB approval and the signing of a data use agreement. Data will only be shared for academic research purposes under conditions that ensure the protection of sensitive information. The extended data in this study are available in the Zenodo repository at https://doi.org/10.5281/zenodo.18521202 ( Pauzan et al., 2024). Data are available under the terms of the Creative Commons Attribution 4.0 International (CC BY 4.0) license.
