# Supplementary material for: Implementation for Code-Switching from the Perspective of Academic Functions in Teaching English at the MAN 1 Praya
Source: F1000Res. 2026 Mar 13;15:397. [Version 1] doi: 10.12688/f1000research.177674.1 (PMC13062769; doi:10.12688/f1000research.177674.1)
Supplement: Peer review history

# Reviewer report: Reviewer response for version 1

**DOI:** 10.5256/f1000research.195931.r470060

**Published:** 2026-04-08

**Authors:** Michael L Estremera

**Affiliations:** 1 Dr. Emilio B. Espinosa Sr. Memorial State College of Agriculture and Technology, Mandaon, Masbate, Philippines

**Introduction**

 Include a theoretical framework that explains where the codeswitching phenomenon fits.

**Methodology**

 Present this part comprehensively by categorizing it into sections such as Research Context, Research Design, Data Collection Procedures, Sampling, Data Analysis and Processing, and Ethical Considerations.

**Results and Discussion**

 Categorize into excerpts (Excerpt 1, 2, 3, 4, and so on) and shorten the classroom transcripts according to academic functions. If possible, please insert pictures of classroom interactions. 

**Conclusion**

 Highlight the novelty claim/s of your study by emphasizing the specific field to which it has contributed. Please also mention the specific theory to which your findings had contributed.

**Reference**

 Cite recent authors from Scopus journals, years 2025 to 2026.

Is the work clearly and accurately presented and does it cite the current literature?

Partly

If applicable, is the statistical analysis and its interpretation appropriate?

Partly

Are all the source data underlying the results available to ensure full reproducibility?

Partly

Is the study design appropriate and is the work technically sound?

Partly

Are the conclusions drawn adequately supported by the results?

Partly

Are sufficient details of methods and analysis provided to allow replication by others?

Partly

Reviewer Expertise:

Language Education, Literature Education

I confirm that I have read this submission and believe that I have an appropriate level of expertise to confirm that it is of an acceptable scientific standard, however I have significant reservations, as outlined above.

# Reviewer report: Reviewer response for version 1

**DOI:** 10.5256/f1000research.195931.r470068

**Published:** 2026-04-01

**Authors:** Olusegun Oladele Jegede

**Affiliations:** 1 Lead City University, Ibadan, Nigeria

Reviewer's Comments

 The background to the study is strong because it clearly defines the area of study and situates code-switching within bilingual and multilingual contexts, but it does not fully connect this practice to its historical or broader social context, which the author should elaborate. Key concepts, definitions, and relevant prior studies are summarised, yet the links between theories and the research focus could be made more explicit. Major debates about code-switching versus code-mixing are identified, though discussion of conflicting perspectives could be expanded. The current context of classroom English learning is explained, but its significance to the research objectives could be emphasised further.

 The research gap is presented clearly, identifying the need to examine classroom code-switching in Indonesian-English contexts. The study is linked to limitations in existing literature, though it could more explicitly specify what prior studies have overlooked. The discussion demonstrates what is known and what is insufficiently explored, but the author should more sharply define the unresolved questions or practical challenges that motivate the study.

 The aim specifies that the study investigates classroom-based code-switching and its functions, highlighting its potential impact on teaching and learning. The objectives are clearly stated, practically oriented, and derived from the aim, but they could be linked more directly to measurable outcomes or specific research questions.

 The study’s qualitative descriptive design is generally appropriate for examining code-switching by English teachers in a multilingual classroom, as it allows detailed, context-rich analysis of classroom interactions. Data collection through observation, recordings, and interviews is suitable, and ethical procedures, including written consent, are properly addressed. However, the study has technical limitations: the sample is limited to two teachers, reducing generalisability; the analytical procedure lacks clarity, with no explicit coding or framework described; and some methodological terms are ambiguous. Strengthening data analysis and providing systematic procedures would enhance the study’s rigor, transparency, and replicability.

 The results section is strong in depth and clarity because it presents detailed classroom interactions and identifies multiple functions of code-switching. The results relate to the study objectives by illustrating how code-switching supports learning and class management. The section lacks a clear introductory paragraph, which the author should include to frame the findings. Data are provided to support the claims, and strong excerpts are used, but some excerpts are only descriptively presented without deeper interpretation. Weak areas include limited critical analysis of broader implications and minimal exploration of cultural meanings in language use. Social, historical, and situational contexts are not fully addressed. Linguistic and rhetorical choices are shown but not thoroughly analysed for effect. Arguments are coherent and mostly supported by evidence, though links to research objectives could be more explicit. Particular attention should be given to interpretation, contextualisation, and analytical depth.

 The discussion could more clearly begin with the study’s purpose. Key findings are summarised but not consistently linked to the research questions. The meaning of the results is interpreted in part, yet some functions are described rather than critically analysed. Comparisons with previous studies are minimal, which the author should address. Evaluation of strengths, limitations, and implications is underdeveloped. The significance of the data is mentioned but not fully contextualised. The discussion constructs a logical argument but lacks depth in insight and contribution to the field. Greater attention is needed on critical evaluation, contextualisation, and connecting findings to broader theoretical and practical implications.

 The conclusions drawn in the study are generally supported by the results, as the detailed examples demonstrate multiple functions of code-switching in classroom communication, both for learning and class management. The data illustrate how teachers clarify content, reinforce concepts, manage student behaviour, provide feedback, motivate learners, and facilitate comprehension through strategic language switching. Each function described in the results is substantiated with actual teacher–student interactions, showing clear links between observed behaviour and the intended instructional or managerial purpose. However, the study is limited to two teachers, which restricts generalisability, and the discussion largely describes occurrences without deeper interpretive analysis of their broader implications.

Is the work clearly and accurately presented and does it cite the current literature?

Yes

If applicable, is the statistical analysis and its interpretation appropriate?

Not applicable

Are all the source data underlying the results available to ensure full reproducibility?

No

Is the study design appropriate and is the work technically sound?

Partly

Are the conclusions drawn adequately supported by the results?

No

Are sufficient details of methods and analysis provided to allow replication by others?

Partly

Reviewer Expertise:

Language of Instruction; Classroom Discourse; Language Challenges in Education

I confirm that I have read this submission and believe that I have an appropriate level of expertise to confirm that it is of an acceptable scientific standard, however I have significant reservations, as outlined above.

# Reviewer report: Reviewer response for version 1

**DOI:** 10.5256/f1000research.195931.r468048

**Published:** 2026-03-27

**Authors:** Alek Alek

**Affiliations:** 1 Syarif Hidayatullah Jakarta Islamic State University, Banten, Indonesia

- Rewrite the 'introduction' section becomes more systematic (context, known, unknown, the significance of the topic or why?, gap, some related previous studies (synthesis) for showing novelty), state of the art, objectives, contribution, and research questions.

 - How did you govern/manage your data procedures?

 - How were your data, method, data analysis procedures guaranteed or validated? Rewrite clearly or rigorly.

 - Use some theories are needed to justify and support your claims or research findings, comparing, inferencing. etc.  

 - Double check the way of presentation of the conclusion. It should be the meaning of the findings and implication, and suggestions.

Is the work clearly and accurately presented and does it cite the current literature?

Yes

If applicable, is the statistical analysis and its interpretation appropriate?

Not applicable

Are all the source data underlying the results available to ensure full reproducibility?

Yes

Is the study design appropriate and is the work technically sound?

Yes

Are the conclusions drawn adequately supported by the results?

Partly

Are sufficient details of methods and analysis provided to allow replication by others?

Yes

Reviewer Expertise:

Pragmatics, Discourse Analysis, applied linguistics

I confirm that I have read this submission and believe that I have an appropriate level of expertise to confirm that it is of an acceptable scientific standard.
